# Corrigendum: Applications of exhaled breath condensate analysis for drug monitoring and bioequivalence study of inhaled drugs

**DOI:** 10.3389/jpps.2023.12042

**Published:** 2023-12-18

**Authors:** Nastaran Hashemzadeh, Elaheh Rahimpour, Abolghasem Jouyban

**Affiliations:** ^1^ Pharmaceutical Analysis Research Center and Faculty of Pharmacy, Tabriz University of Medical Sciences, Tabriz, Iran; ^2^ Research Center for Pharmaceutical Nanotechnology, Tabriz University of Medical Sciences, Tabriz, Iran; ^3^ Infectious and Tropical Diseases Research Center, Tabriz University of Medical Sciences, Tabriz, Iran; ^4^ Pharmaceutical Sciences Research Center, Shahid Beheshti University of Medical Sciences, Tehran, Iran

**Keywords:** exhaled breath condensate, bioequivalence, pharmacodynamic, pharmacokinetic, inhalers

In the original article, there was a mistake in Table 1 as published. The order of the titles of the 2nd and 3rd columns have been reversed. The corrected form is that the title of the 2nd column is read as “EBC Conc.”, and that of the 3rd column as “Plasma Conc.”. The corrected column titles are marked with red font in the following Table.

**TABLE 1:** An overview of drug concentrations in EBC and plasma samples, along with some details of the determination procedures.

Drug	EBC Conc.	Plasma Conc.	Analytical platform for EBC samples	LR^1^/LOD^2^ for EBC samples	Significant feature	References
Alprazolam	NR^3^	0.005–0.02 ppm	LC^4^–MS^5^	2–18/1 pg. filter^1^	Sensitive	(30, 40)
Amikacin	(0.42–0.68) × 10^−3^ ppm	1.91–2.81 ppm	HPLC^6^–MS/MS	0.21–3,000/0.06 × 10^−3^ ppm	Quick and efficient	(43)
Amphetamine	NR^3^	0.02–0.15 ppm	LC–MS	2–18/3 pg. filter^1^	Sensitive	(30, 40)
Aspirin	23.2–24.9 ppm	150–300 ppm	Colorimetry	10–250/4.1 ppm	High reliability	(41, 42)
Benzoylecgonine	NR^3^	0.018–0.14 ppm	LC–MS	2–18/0.5 pg. filter^−1^	Sensitive	(30, 40)
Buprenorphine	NR^3^	0.001–0.005 ppm	LC-MS/MS	NR^3^/2.5 × 10^−3^ ppm	Non-invasive and useful	(14, 40)
Buprenorphine	NR^3^	0.001–0.005 ppm	LC–MS	2–18/2 pg. filter^−1^	Sensitive	(30, 40)
Carbamazepine	0.3–0.5 ppm	2–12 ppm	Spectroflourimetry	0.2–20/0.08 ppm	Sensitive	(15, 40
Cocaine	NR^3^	0.1–0.3 ppm	LC–MS	2–18/2 pg. filter^−1^	Sensitive	(30, 40)
Codeine	NR^3^	0.025–0.25 ppm	LC-MS/MS	NR^3^/0.1 × 10^−3^ ppm	Non-invasive and useful	(14, 40)
Daclatasvir	0.048–0.992 ppm	0.052–0.852 ppm	Plasmon resonance	0.01–1.0/0.008 ppm	Low LOD, low cost, sensitive	(44, 45)
Daclatasvir	NR^3^	0.052–0.852 ppm	Spectroflourimetry	0.5–15 × 10^−3^/0.12 × 10^−3^ ppm	Simple, fast and sensitive	(46, 45)
Deferiprone	0.06–0.17 ppm	5–25 ppm	Spectroflourimetry	0.06–1.50/0.06 ppm	Simple, low EBC volume	(21, 47)
Diazepam	NR^3^	0.2–2 ppm	LC–MS	2–18/1 pg. filter^−1^	Sensitive	(30, 40)
Doxorubicin	(48.9–203) × 10^−3^ ppm	0.006–0.09 ppm	Spectrophotometric	0.02–0.2/0.00416 ppm	Simple, sensitive and reliable	(24, 40)
2-Ethylidene-1,5-dimethyl-3,3-diphenylpyrrolidine	NR^3^	NR^3^	LC-MS/MS	NR^3^/0.01 × 10^−3^ ppm	Non-invasive and useful	(14, 40)
Fentanyl	NR^3^	0.005–0.3 ppm	LC-MS/MS	NR^3^/0.05 × 10^−3^ ppm	Non-invasive and useful	(14, 40)
Hydromorphone	NR^3^	0.001–0.03 ppm	LC-MS/MS	NR^3^/1 × 10^−3^ ppm	Non-invasive and useful	(14, 40)
Hydrocodone	NR^3^	0.01–0.1 ppm	LC-MS/MS	NR^3^/0.5 × 10^−3^ ppm	Non-invasive and useful	(14, 40)
Lamotrigine	0.592–0.771 ppm	3–15 ppm	Spectrophotometric	NR^3^/0.005 ppm	Quick visual detection	(28, 40)
Lamotrigine	0.55–1.19 ppm	3–15 ppm	Spectroflourimetry	0.05–2.0/0.011 ppm	Sensitive and fast	(48, 40)
Meperidine	NR^3^	0.1–0.8 ppm	LC-MS/MS	NR^3^/0.05 × 10^−3^ ppm	Non-invasive and useful	(14, 40)
Meropenem	Not detectable	25.5 ppm	UHPLCHR-MS	21,168 pg. filter^−1^/NR^3^	Non-invasive	(55, 56)
Methadone	NR^3^	0.05–0.5 ppm	LC-MS/MS	NR^3^/0.5 × 10^−3^ ppm	Non-invasive and useful	(14, 40)
Methadone	0.16–1.06 ppm	0.05–0.5 ppm	Capillary electrophoresis	0.15–5 ppm/0.15 ppm	Simple, sensitive and accurate	(16, 40)
Methadone	23.6–275 pg.min^−1^	0.05–0.5 ppm	LC–MS–MS	100–2000/3 pg/sample	Feasible	(12, 40)
Methadone	(0.34–1.31) × 10^−3^ ppm	0.05–0.5 ppm	LC	0.5–10 × 10^−3^/0.5 × 10^−3^ ppm	Simple and low cost	(23, 40)
Methadone	0.7–0.48 ppm	0.05–0.5 ppm	Capillary electrophoresis	0.3–5/0.3 ppm	Simple and fast	(26, 40)
Methadone	NR^3^	0.05–0.5 ppm	LC–MS	2–18/0.5 pg. filter^−1^	Sensitive	(30, 40)
Methamphetamine	NR^3^	0.01–0.05 ppm	LC–MS	2–18/1 pg. filter^−1^	Sensitive	(30, 40)
Methotrexate	(45.4–140.8) × 10^−3^ ppm	2.27 ppm	Spectrofluorimetry	20–998.8 × 10^−3^/15.9 × 10^−3^ ppm	Simple, fast and accurate	(40, 49)
Metoprolol	NR^3^	0.02–0.5 ppm	Spectrofluorimetry	5−100 × 10^−3^/2.1–3.4 × 10^−3^ ppm	Simple, low-cost	(40, 50)
6-Acetyl morphine	NR^3^	0.015–0.10 ppm	LC–MS	2–18/1 pg. filter^−1^	Sensitive	(30, 40)
Morphine	NR^3^	0.01–0.15 ppm	LC–MS	2–18/1 pg. filter^−1^	Sensitive	(30, 40)
Morphine	(0.10–5.48) × 10^−3^ ppm	0.01–0.15 ppm	LC-MS/MS	NR^3^/0.1 × 10^−3^ ppm	Non-invasive and useful	(14, 40)
Morphine	(89–173) × 10^−3^ ppm	0.01–0.15 ppm	GC^7^-MS	NR^3^/2.1 × 10^−3^ ppm	Repeatable and stable	(20, 40)
Naloxone	NR^3^	0.01–0.03 ppm	LC-MS/MS	NR^3^/0.25 × 10^−3^ ppm	Non-invasive and useful	(14, 40)
Naltrexone	NR^3^	0.005–0.03 ppm	LC-MS/MS	NR^3^/0.5 × 10^−3^ ppm	Non-invasive and useful	(14, 40)
Oxazepam	NR^3^	0.2–1.5 ppm	LC–MS	2–18/1 pg. filter^−1^	Sensitive	(30, 40)
Oxycodone	NR^3^	0.02–0.05 ppm	LC-MS/MS	NR^3^/0.25 × 10^−3^ ppm	Non-invasive and useful	(14, 40)
Oxymorphone	NR^3^	NR^3^	LC-MS/MS	NR^3^/0.75 × 10^−3^ ppm	Non-invasive and useful	(14, 40)
Oxymorphone	(29–82) × 10^−3^ ppm	NR^3^	GC-MS	NR^3^/1.5 × 10^−3^ ppm	Repeatable, and stable	(20, 40)
Paracetamol	1.12–4.68 ppm	2.5–25 ppm	Colorimetry	0.2–10.0/0.49 ppm	Specific and simple	(17, 40)
Phenobarbital	0.21–1.65 ppm	1–5 ppm	Spectrofluorimetry	0.1–10.0/0.024 ppm	Feasible, efficient and simple	(40, 51)
Phenobarbital	0.72–1.80 ppm	1–5 ppm	Spectrofluorimetry	0.01–8.0/0.006 ppm	Reliable and sensitive	(40, 52)
Phenytoin	0.013–0.13 ppm	5–20 ppm	Capillary electrophoresis	0.001–0.10/0.001 ppm	Selectivity	(40, 53)
Piperacillin	90 × 10^−3^ ppm	5–20 ppm	Microfluidic sensor	NR^3^/56 × 10^−3^ ppm	Versatile and low LOD	(39, 40, 54)
Piperacillin	45 pg	5–20 ppm	UHPLCHR^8^-MS	988–203,895/3,083 pg.filter^−1^	Non-invasive	(55, 56)
Propranolol	0.030 ppm	0.02–0.3 ppm	LC-MS/MS	5.6–224 × 10^−3^ ppm/NR^3^	Simple, cheap and feasible	(31, 40)
Tazobactam	90 × 10^−3^ ppm	7.7–13.7 ppm	Microfluidic sensor	NR^3^/56 × 10^−3^ ppm	Versatile and low LOD	(39, 40, 54)
Tazobactam	45 pg	7.7–13.7 ppm	UHPLCHR^8^-MS	988–203,895/3,083 pg. filter^−1^	Non-invasive	(55, 56)
Tetrahydrocannabinol	NR^3^	0.001–0.007 ppm	LC–MS	2–18/3 pg. filter^1^	Sensitive	(30, 40)
Tobramycin	(13.7–32.2) × 10^−3^ ppm	5–10 ppm	Colorimetry	1.0–50.0 × 10^−3^/0.5 × 10^−3^ ppm	Repeatable and low LOD	(18, 40)
Tobramycin	(21.4–41.6) × 10^−3^ ppm	5–10 ppm	UV spectroscopy	1.0–50.0 × 10^−3^/(0.5 × 10^−3^ ppm	Sensitive	(13, 40)
Tobramycin	(2.4–17.0) × 10^−6^ ppm	5–10 ppm	LC–MS	NR^3^	Wide LR	(32, 40)
Tramadol HCl	NR^3^	0.1–1 ppm	LC-MS/MS	NR^3^/0.5 × 10^−3^ ppm	Non-invasive and useful	(14, 40)
Salbutamol	(32.2–645.0) × 10^−6^ ppm	<0.01–0.02 ppm	LC–MS	NR^3^	Wide LR	(32, 40)
Salbutamol sulfate	(89–173) × 10^−3^ ppm	<0.01–0.02 ppm	GC-MS	0.615–5/370 ppm	Wide LR and low LOD	(40, 57)
Valproic acid	(0.13–500) × 10^−3^ ppm	40–100 ppm	GC-MS	1.0–5.0 × 10^−3^/0.08 × 10^−3^ ppm	Repeatable, wide LR	(27, 40)
Vancomycin	0.36–1.87 ppm	5–40 ppm	Spectrofluorimetry	0.1–8/0.06 ppm	Sensitive and low cost	(19, 40)
Verapamil	0.059–0.067 ppm	0.05–0.25 ppm	Spectrofluorimetry	0.02–12.0/0.008 ppm	Suitable and accurate	(29, 40)

LR^1^: Linear range; LOD^2^: Limit of detection; NR^3^: Not reported; LC^4^: Liquid chromatography; MS^5^: Mass spectrometry; HPLC^6^: High-performance liquid chromatography; GC^7^: Gas chromatography; UHPLCHR^8^: Ultra-high-pressure liquid chromatography high-resolution mass spectrometry.

The authors apologize for this error and state that this does not change the scientific conclusions of the article in any way.

